# Molecular Characterization and Functional Analysis of Leucine Zipper Transcription Factor Like 1 in Zebrafish (*Danio rerio*)

**DOI:** 10.3389/fphys.2019.00801

**Published:** 2019-06-25

**Authors:** Qun Wei, Yongxia Chen, Yi-Feng Gu, Wenhe Zhao

**Affiliations:** ^1^Department of Surgical Oncology, Institute of Clinical Medicine, Sir Run Run Shaw Hospital, School of Medicine, Zhejiang University, Hangzhou, China; ^2^State Key Laboratory for Diagnosis and Treatment of Infectious Diseases, Collaborative Innovation Center for Diagnosis and Treatment of Infectious Diseases, The First Affiliated Hospital, School of Medicine, Zhejiang University, Hangzhou, China

**Keywords:** LZTFL1, HH, tumor suppressor, sequence analysis, zebrafish

## Abstract

Leucine zipper transcription factor like 1 (LZTFL1) is a member of the Bardet–Biedl syndrome gene family. LZTFL1-null mice show the phenotype of obesity, retinal degeneration, and abnormal cilia development. Functionally, LZTFL1 serves as a tumor suppressor and a negative regulator in the hedgehog signaling pathways. The biological function of mammalian LZTFL1 is partially addressed, but data on other model organisms are limited. Zebrafish (*Danio rerio*) is widely considered as a powerful model to understand the functions of genes implicated in obesity, disease, and cancer. In this study, LZTFL1 homologs were identified in zebrafish (zebrafish LZTFL1). The full-length cDNA of zebrafish LZTFL1 contained 897 bps encoding 298 amino acids. Zebrafish LZTFL1 displayed conserved domains of coil–coil and leucine zipper domain. PCR results showed that zebrafish LZTFL1 was widely distributed in various tissues. Western blot analysis further revealed that zebrafish LZTFL1 was detected to be ectopically expressed in HeLa cells with correct molecular weight. Fluorescence images showed as well that zebrafish LZTFL1 was localized in the cytoplasm. Furthermore, luciferase reporter assay indicated zebrafish LZTFL1 served as a negative regulator in the hedgehog signaling pathway. These data supported that zebrafish was a good model for understanding the biological roles of LZTFL1.

## Introduction

Leucine zipper transcription factor like 1 (LZTFL1), also called BBS17, is a member of the Bardet–Biedl syndrome (BBS) gene family (Sakurai et al., 2011; Khan et al., 2016). BBS is a human genetic disorder correlated with ciliary dysfunction (Seo et al., 2011; Marion et al., 2012; Novas et al., 2015; Suspitsin and Imyanitov, 2016). Clinically, BBS patients are characterized with non-sense mutations of LZTFL1/BBS17 display obesity, polydactyly, diabetes, renal abnormalities, hypogenitalism, hypertension, cognitive impairment, and retinal degeneration (Schaefer et al., 2014; Novas et al., 2015; Khan et al., 2016).

Human LZTFL1 is located ∼5 Mb from the LUCA region on the telomeric end of the 3p21.3 region (Kiss et al., 2001), a hotspot for tumor-suppressor genes (Ji et al., 2005; Hesson et al., 2007). LZTFL1 mRNA and protein are widely distributed in various human and mouse tissues. Functionally, LZTFL1 was first confirmed to be a tumor suppressor (Wei et al., 2010). Immunohistochemistry results indicated that LZTFL1 was highly expressed in the epithelial cells of normal tissues and showed significantly lower expression in the corresponding tumor samples including breast, liver, ovary, stomach, lung, and thyroid cancer (Wei et al., 2010; Wang et al., 2014). Clinically, LZTFL1 expression level displayed significant correlation with longer survival time of cancer patients and had significantly inverse correlation with tumor metastasis (Wei et al., 2010, 2015; Wang et al., 2014). The ectopic expression of LZTFL1 in tumor cells largely reduced the anchorage-independent cell growth and cell migration *in vitro* and inhibited tumor growth *in vivo*. Colocalization studies illustrated that the expression of LZTFL1 overlapped with that of E-cadherin at the plasma membrane in differentiated normal colonic epithelial cells, and this colocalization was absent in colorectal carcinomas due to the loss of LZTFL1 protein expression (Wei et al., 2010). The colocalization of LZTFL1 with E-cadherin suggested that LZTFL1 may stabilize E-cadherin-mediated adherens junction (Wei et al., 2010; Wang et al., 2014). The re-constitution of LZTFL1 in lung tumor cells suppressed the extravasation/colonization of circulating tumor cells into the lung and inhibited tumor growth *in vivo* (Wei et al., 2015). Mechanistically, inhibition of lung cancer metastasis by LZTFL1 may have resulted from two possibilities. First, LZTFL1 may suppress the epithelial-to-mesenchymal transition via the inhibition of the MAPK signaling pathways. Second, LZTFL1 may promote the maintenance of the differentiated state of lung epithelial cells via the suppression of the hedgehog signaling (HH) pathways (Wei et al., 2015).

LZTFL1 is a BBSome interacting protein. LZTFL1 serves as a negative regulator affecting BBSome ciliary trafficking and LZTFL1 dysfunction results in compensation for the loss of certain BBS proteins and the restoration of BBSome ciliary trafficking (Seo et al., 2011). Furthermore, BBSome and LZTFL1 regulate the ciliary trafficking of Smoothened, a 7-transmembrane hedgehog signal transducer. Therefore, LZTFL1 suppresses the ciliary entry of BBSome that is associated with the ciliary trafficking of SMO, making it a negative regulator of HH pathway (Corbit et al., 2005; Ocbina and Anderson, 2008; Kim et al., 2009). HH pathway has been implicated in organizing the body plan, organogenesis, and developing various tumors (Maitah et al., 2011; Park et al., 2011; Jia et al., 2015; Kugler et al., 2015).

LZTFL1 also modulates T cell activation and enhances IL-5 production. The ectopic expression of LZTFL1 in CD4+ T cells increased the T cell activation signal, possibly through LZTFL1-induced TCR–NFAT signaling. Moreover, LZTFL1 silencing reduced Th2 cytokine production, especially IL-5 mRNA and protein production, and further suppressed ATRA-induced IL-5 production (Jiang H. et al., 2016).

Recently, LZTFL1 knockout mice was well established, which showed the phenotype of obesity, retinal degeneration, and abnormal cilia development (Datta et al., 2015; Jiang J. et al., 2016; Wei et al., 2018). Also, zebrafish is a well-established genetic model system for development, immunology, homeostasis, and cancer study (Amole and Unniappan, 2009; Cleal and Parker, 2018; Elbialy et al., 2018; Gehrig et al., 2018), but LZTFL1 study in zebrafish is limited. In this study, we found and cloned one LZTFL1 homolog in zebrafish. Zebrafish LZTFL1 displayed a “leucine zipper pattern” feature, which is characteristically conserved among vertebrates LZTFL1. Further experiment confirmed that zebrafish was a negative regulator in the HH pathway. This study will contribute to a better understanding of LZTFL1 biological function by using zebrafish model.

## Materials and Methods

### Experimental Fish

Two-month-old zebrafish, *Danio rerio*, of both sexes, were kept in running water at 28°C and fed commercial food twice a day. The fish were maintained in the laboratory for at least 2 weeks prior to experimental use to allow the acclimatization and evaluation of their overall health. Only healthy fish, as determined by general appearance and level of activity, were used for these studies. All animal experimental procedures were performed in accordance with the Regulations for the Administration of Affairs Concerning Experimental Animals approved and authorized by Zhejiang University (Document ID: 2018320).

### Molecular Cloning of Zebrafish LZTFL1 cDNA and Tissue Distribution

The sequence of LZTFL1 was initially searched on the zebrafish genome database of UCSC by using the human LZTFL1 amino acid sequence (NP_065080.1) as a probe. Aided by various software programs such as Genscan and BLAST, a possible zebrafish homology of human LZTFL1 was compiled, which was subsequently used for PCR primer design.

Zebrafish were sacrificed after administration of anesthesia, and total RNA was extracted by using TRIzol reagent (Invitrogen) from zebrafish liver and then treated with RNase-free DNase I (Qiagen). The first-strand cDNA was transcribed using an RT-PCR kit (Invitrogen) according to the manufacturer’s instructions. The full-length cDNA of zebrafish LZTFL1 (zebrafish LZTFL1) was amplified using specific primers, 5′-TTT GGA TCC ATG GCT GAT TTT GGT TTT AAT-3′ (forward primer) and 5′-AAA CTC GAG CGC TCA GCT GAT TCA TAT CTT AG-3′ (reverse primer). PCR amplicons were purified using a gel extraction kit (Qiagen) and then ligated into the pGEM-T EASY vector (Promega) for competent cell transformation. Positive clones were sent out for sequencing.

To further validate zebrafish LZTFL1 mRNA distribution, we extracted total RNA rom various tissues, including kidney, liver, gill, skin, brain, intestine, spleen, and muscle. The tissue distribution of zebrafish LZTFL1 mRNA was validated using forward primer, 5′-TTT GGA TCC ATG GCT GAT TTT GGT TTT AAT-3′, and reverse primer, 5′-AAA CTC GAG CGC TCA GCT GAT TCA TAT CTT AG-3′. Also, β-actin mRNA was amplified as an internal control by using forward primer, 5′- ACA CCT TCT ACA ATG AGC TG, and reverse primer, 5′-CTG CTT GCT GAT CCA CAT CT-3′.

### Sequence and Phylogenetic Analysis of Zebrafish LZTFL1

Multiple alignments were performed with the ClustalX program 1.83. The functional domain was validated by Pfam^1^. The phylogenetic tree was constructed by the neighbor-joining method with MEGA 6.0 for 1000 replicates (Saitou and Nei, 1987; Kumar et al., 2004).

### Plasmid Construction, Transfection, and Luciferase Assay

The full-length cDNA of zebrafish or human LZTFL1 was cloned into pcDNA6/myc-HisB (Invitrogen) between the BamHI and XhoI site to construct eukaryotic expression vectors named pcDNA6-Myc-ZeLZTFL1 or pcDNA6-Myc-HuLZTFL1 in which LZTFL1s were fused with Myc-tag in the C terminus.

HeLa cells (ATCC catalog number: CCL-2) and Light2 cells (ATCC catalog number: JHU-68), which were derived from NIH/3T3 cell line transfected with Gli1-responsive Firefly luciferase reporter and a constitutive Renilla-luciferase expression vector were maintained in DMEM supplemented with 10% FBS, penicillin (100 U/mL), and streptomycin (100 μg/mL) at 37°C in 5% CO_2_. HeLa cells were transfected with empty vector pcDNA6-Myc or pcDNA6-Myc-ZeLZTFL1/pcDNA6-Myc-HuLZTFL1 by Liopofectamine 2000 following the manufacturer’s protocol. At 48 h post-transfection, the transfected cells (HeLa-EV, HeLa-ZeLZTFL1, and HeLa -HuLZTFL1) were harvested for further experiment.

To elucidate zebrafish LZTFL1 roles in the hedgehog pathway, Light2 cells were co-cultured alone, with transfected cells HeLa-EV, HeLa-HuLZTFL1, or HeLa-ZeLZTFL1 for 48 h. Then, Light2 cells were lysed with Reporter Lysis Buffer (Promega) for measuring luciferase activity by using Dual-Luciferase Assay Reagent (Promega).

### Western Blot and Immunofluorescence

pcDNA6-Myc-ZeLZTFL1 was transfected into HeLa cells. At 48 h post-transfection, transfected cells were lysed for Western blot analysis. Equal amounts of protein fraction were loaded on 12% SDS-PAGE gels, separated, and electrophoretically transferred into a PVDF membrane. The membrane was blocked with 5% BSA for 30 min, incubated with rabbit anti-MYC antibodies (Cell Signaling #2278, 1:2000 dilution) for 2 h, and incubated with goat anti-Rabbit IgG (Thermo #31460, 1:5000) at room temperature for 1 h. The membrane was washed thrice with TBST buffer (25 mM Tris–HCl pH 7.5, 150 mM NaCl, and 0.1% Tween 20) and developed with ECL solution. The membrane was also incubated with anti-tubulin antibodies of loading control (Cell Signaling #2144, 1:5000 dilution).

For immunofluorescent staining, pcDNA6-Myc-ZeLZTFL1 was transfected into HeLa cells for 48 h. The transfected cells were fixed with 3% (v/v) formaldehyde for 10 min and permeabilized with 0.2% Triton X-100 in PBS for 15 min. The fixed cells were blocked with 5% (v/v) goat serum at 37°C for 1 h, which were then incubated with rabbit anti-MYC antibodies at 4°C overnight and then with FITC-labeled goat anti-rabbit IgG secondary antibodies at 37°C for 1 h. Furthermore, the cells were stained with DAPI (nuclei marker) for 1 min, and cell subcellular localization images were captured under a fluorescent microscope.

### Statistical Analysis

Variations between experimental groups were statistically analyzed using ANOVA and multiple Student’s *t*-tests. Statistical significance was set at ^∗^*p* < 0.05 and ^∗∗^*p* < 0.01. Data points were analyzed from at least three independent experiments. All mean values were expressed with the standard deviation.

## Results

### Molecular Characterization of Zebrafish LZTFL1

**FIGURE 1:**
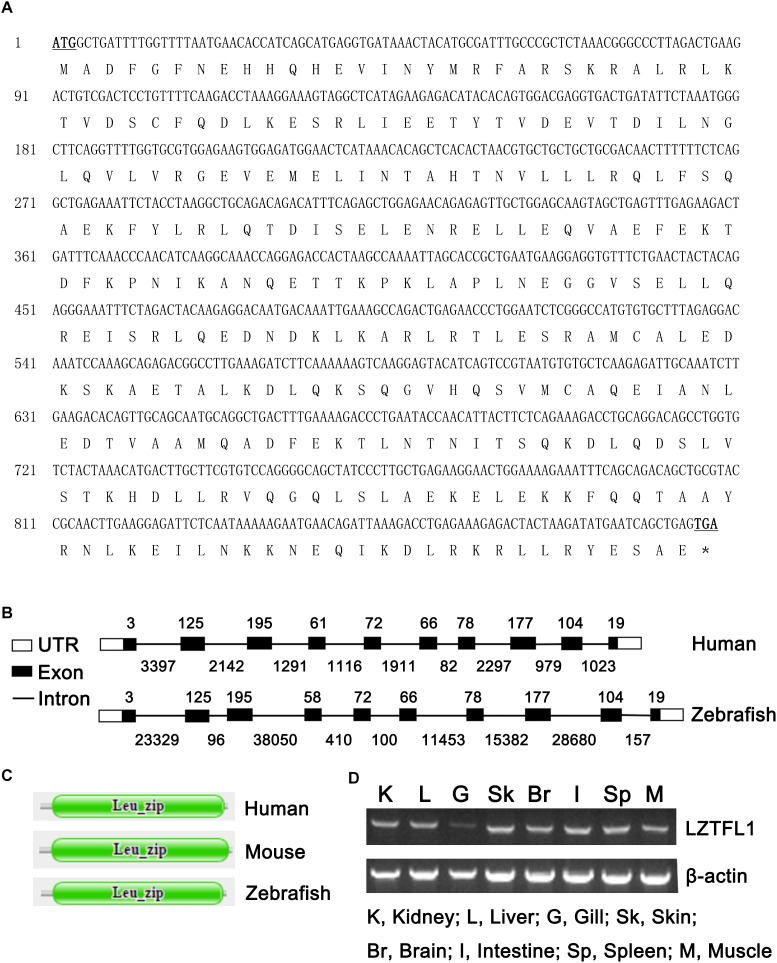
**(A)** Nucleotide and deduced amino acid sequences of LZTFL1 cDNA from zebrafish (GenBank accession number MK253678). The start and stop codons are underlined and in boldface. ^∗^stop codon. **(B)** Genomic structures of LZTFL1 genes. The rectangles represent the exons, and the lines between indicate the introns. The sizes of exons are marked above the exons, and the sizes of introns are marked below the introns. White boxes indicate 5′ or 3′ untranslated region (UTR), and black boxes indicate the encoding-exons. **(C)** Schematic representation of the domain organization of LZTFL1 proteins. Each domain was predicted by the Pfam programs. **(D)** Zebrafish LZTFL1 was distributed in various tissues, including kidney, liver, gill, skin, brain, intestine, spleen, and muscle.

**FIGURE 2:**
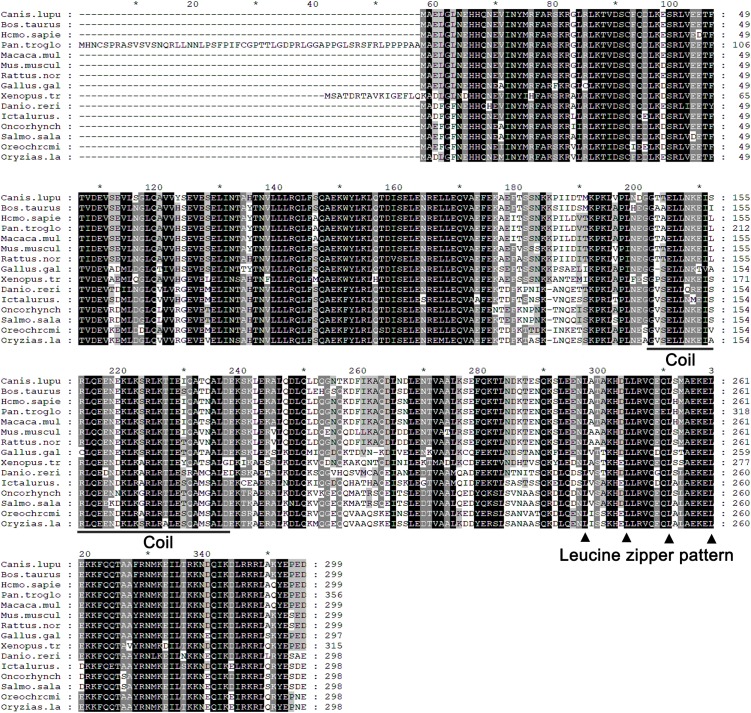
Alignment of the predicted amino acid sequence of LZTFL1 genes among different species. Residues shaded in black are completely conserved across all species, and residues shaded in gray are similar with respect to the side chains. The dashes in the amino acid sequences indicate gaps introduced to maximize the alignment. The putative coil–coil domain was underlined. Four leucine residues, which constituted the “leucine zipper pattern,” were indicated by black triangle.

The full-length cDNA of zebrafish LZTFL1 (GenBank accession number MK253678) is 897-bp-long, which encodes a putative protein of 298 amino acids (Figure 1A) with theoretical molecular weight of 34.468 kDa and isoelectric point of 5.71. Multiple amino acid alignment of LZTFL1 encompassing representatives of various species, including primate, rodent, bird, frog, and teleost fish, was performed using ClustalW. As shown in Figure 2, the putative domains of coil–coil and leucine zipper were revealed in zebrafish LZTFL1, displaying high homology among vertebrates. The conserved feature was found in the signature of four leucine residues in the C-terminal of zebrafish LZTFL1 (Figure 2), which constituted the “leucine zipper pattern” among vertebrates (Figure 1C).

Zebrafish LZTFL1 gene is located within a 118 554-bp genomic fragment on chromosome 24. The intron-splicing consensus (GT/AG) was constant at the 5′ and 3′ ends of the introns in zebrafish LZTFL1 gene. Similar to human LZTFL1, zebrafish LZTFL1 gene has 10 encoding-exons and the size and organization of all exons in LZTFL1 genes matches well between human and zebrafish (Figure 1B). However, the length of zebrafish LZTFL1 gene (118 554 bp long) is dramatically longer than that of human LZTFL1 gene (only 15 578 bp long), because the intron size of zebrafish LZTFL1 gene notably differs a lot from that of human gene (Figure 1B).

### Multiple Sequence Alignment and Phylogenetic Analysis

**FIGURE 3:**
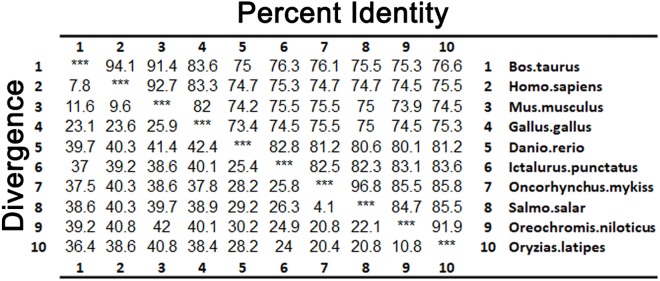
Percentages (%) of amino acid sequence identity for LZTFL1 sequences.

**FIGURE 4:**
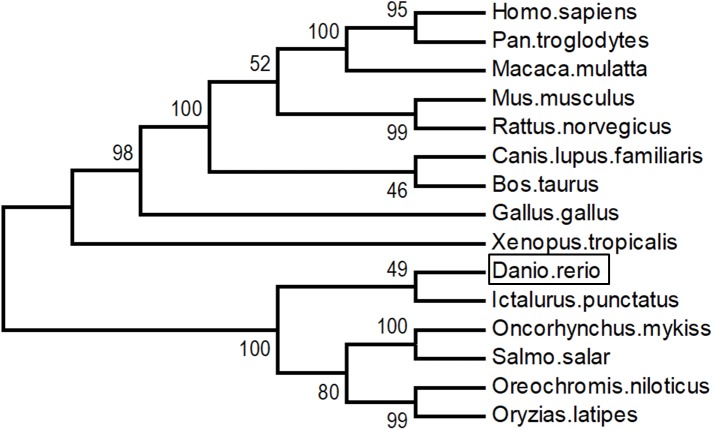
Unrooted phylogenetic tree of LZTFL1 protein was constructed using the neighbor-joining method by Mega 6.0. Node values represent the percent bootstrap confidence derived from 1000 replicates.

As shown in Figure 3, LZTFL1s display 80.1–82.8% identity among fish species, 74.2–75% between fishes and mammals, and 73.4% between fishes and birds. The phylogenetic analysis tree constructed by neighbor-joining method indicates that zebrafish LZTFL1 merged into the teleost subgroup, and amphibians, birds, and mammalian homologs were clustered into their corresponding subgroups (Figure 4).

### LZTFL1 Tissue Distribution

RT-PCR was conducted to validate the expression patterns of zebrafish LZTFL1 in various tissues of zebrafish. Results showed that zebrafish LZTFL1 mRNA was widely distributed in kidney, liver, gill, skin, brain, intestine, spleen, and muscle (Figure 1D).

### Subcellular Localization of Zebrafish LZTFL1

**FIGURE 5:**
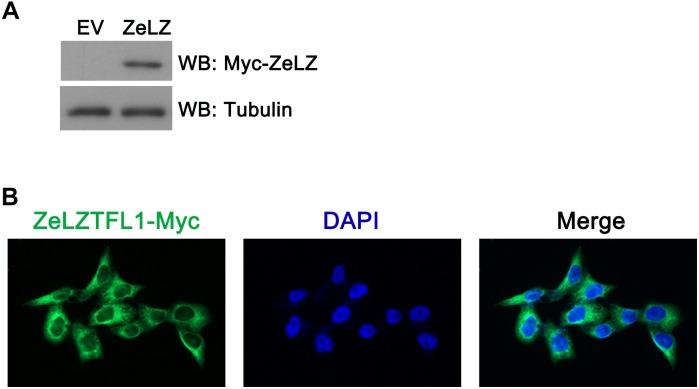
**(A)** Myc-tagged zebrafish LZTFL1 plasmids were transfected into HeLa cells. At 48 h post-transfection, cells were lysed for Western blot analysis. As shown in the picture, compared with empty vectors, LZTFL1 fused with Myc tag was ectopically expressed in HeLa cells. **(B)** Myc-tagged zebrafish LZTFL1 was transfected into HeLa cells. At 48 h post-transfection, cells were fixed and probed with FITC-anti-Myc antibodies and DAPI (nuclear counterstain). Moreover, zebrafish LZTFL1 showed predominant localization in the cytoplasm but subtle localization in nuclei. Magnification, 200×. EV, empty vector, pcDNA6-Myc; ZeLZ, pcDNA6-Myc-ZeLZTFL1, zebrafish LZTFL1 was inserted into pcDNA6-Myc.

To investigate the subcellular localization of zebrafish LZTFL1, we transfected Myc-tagged zebrafish LZTFL1 (pcDNA6-myc-ZeLZTFL1) into HeLa cells. At 48 h post-transfection, HeLa cells were lysed for Western blot analysis. As shown in Figure 5A, the expression of Myc-tagged zebrafish LZTFL1 was detected by Western blot analysis with the correct molecular weight of approximately 35 kDa. Moreover, fluorescence images illustrated that FITC-anti-Myc-labeled zebrafish LZTFL1 was mainly distributed in the cytoplasm and cell membrane but subtly in the nuclei with similar subcellular localization to that of mammalian LZTFL1 (Figure 5B).

### Zebrafish LZTFL1 Suppressing Hedgehog Signaling

**FIGURE 6:**
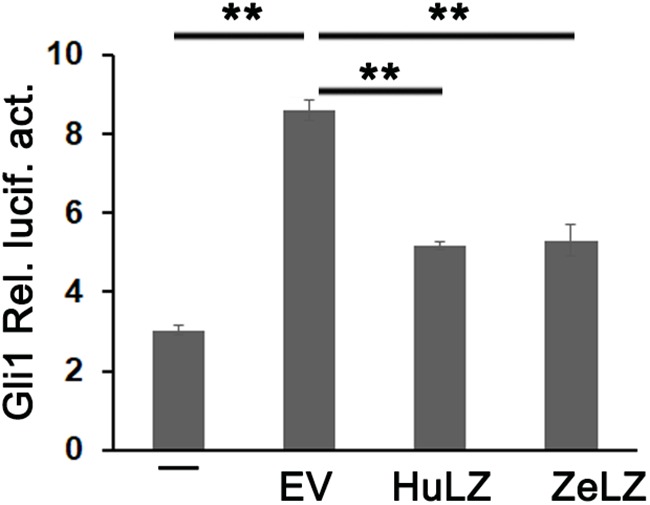
Gli1-luc reporter activities from HH reporter cell line Light2 cultured alone (–) with HeLa-EV (EV, empty vector, pcDNA6-Myc), HeLa-HuZeLZTFL1 (pcDNA6-Myc-HuLZTFL1), or HeLa-ZeLZTFL1 (pcDNA6-Myc-ZeLZTFL1). Luciferase reporter assay indicated that the LZTFL1 of both human and zebrafish can suppress HH signaling. Each bar represents the mean ± SEM of at least five samples. The asterisks denote statistically significant differences between the indicated samples. ^∗∗^*p* < 0.01. ZeLZ, pcDNA6-Myc-ZeLZTFL1 (zebrafish LZTFL1 was inserted into pcDNA6-Myc). HuLZ, pcDNA6-Myc-HuLZTFL1 (human LZTFL1 was inserted into pcDNA6-Myc).

Mammalian LZTFL1 affected cilia development and trafficking and disrupted hedgehog signaling. In general, Light2 cells with stably transfected Gli1-luciferase reporter were activated in response to hedgehog signaling. To detect whether zebrafish LZTFL1 suppressed hedgehog signaling, we transfected empty vector (EV) or ectopic expression plasmids of LZTFL1 cDNA (HuLZTFL1, human LZTFL1 or ZeLZTFL1, zebrafish LZTFL1) into HeLa cells, and then EV- or LZTFL1-transfected HeLa cells (HeLa-EV, HeLa-HuZeLZTFL1, or HeLa-ZeLZTFL1) were co-cultured with Light2 cells. Light2 cells were lysed for luciferase assay, and reporter assay results indicated that compared with the control group with HeLa-EV/Light2 co-culture, Gli1-luciferase activity was significantly decreased in HeLa-ZeLZTFL1/Light2 co-culture similar to that in positive control HeLa-HuLZTFL1/Light2 co-culture, suggesting that the hedgehog secreted by HeLa-ZeLZTFL1 cells was reduced (Figure 6).

## Discussion

LZTFL1 was initially identified as a tumor suppressor (Wei et al., 2010). The low expression of LZTFL1 was correlated with the tumor grades and metastasis and predicted poor patient outcome (Wang et al., 2014). LZTFL1 re-constitution in tumor cells suppressed the growth of tumor cells derived graft *in vivo* and inhibited the extravasation/colonization of tumor cells into the lung (Wei et al., 2015). LZTFL1 served as a specific regulator of BBSome ciliary trafficking. LZTFL1 inhibited ciliary entry of BBSome, which was involved in the ciliary trafficking of SMO. Therefore, LZTFL1 was a negative regulator of the HH pathway (Seo et al., 2011). Furthermore, LZTFL1 biological function was characterized in LZTFL1-null mice. LZTFL1-null mice displayed pleiotropic phenotypes, which are commonly observed in BBS, including retinal degeneration and obesity (Datta et al., 2015; Jiang J. et al., 2016; Wei et al., 2018). Mechanistically, the obese phenotype of LZTFL1-null mice was due to the LZTFL1 loss in the brain. LZTFL1-null mice were resistant to leptin. LZTFL1 silencing decreased STAT3 phosphorylation in the leptin receptor signaling pathway in the hypothalamus upon leptin stimulation. Besides, LZTFL1-deficient mouse embryonic fibroblasts (MEFs) significantly displayed longer cilia than wild-type MEFs (Wei et al., 2018).

To some extent, LZTFL1 roles in mammalian was partially addressed, but limited data are available about the LZTFL1 function in zebrafish. The zebrafish is a powerful model organism for the study of vertebrate biology, being well suited to both developmental and human disease such as obesity, diabetes, and cancer (Elbialy et al., 2018; Enya et al., 2018; Gehrig et al., 2018; Gore et al., 2018). In the current study, the full-length cDNA of LZTFL1 was identified, which was 897-bp-long with a prediction of 299 amino acids. The size and number of coding exons within LZTFL1 between human and zebrafish are almost the same. Surprisingly, zebrafish LZTFL1 gene within genome distributed at a 118 554-bp genomic fragment, which is much longer than that human LZTFL1 gene, was located within 15 578 bp in the genome due to the high variation of intron size of LZTFL1 genes between zebrafish and human.

Multiple sequence alignment and BLAST analysis confirmed that LZTFL1 homolog in zebrafish contained conserved leucine zipper and coil–coil domain. Similar to mammalian LZTFL1s, zebrafish LZTFL1 was characterized with typical “leucine zipper pattern” with four leucine residues in the C-terminal (Kiss et al., 2001). Hence, zebrafish LZTFL1 may potentially play roles in the HH pathway such as human LZTFL1.

To investigate potential zebrafish LZTFL1 function, we ectopically expressed HeLa cells with Myc-tagged zebrafish LZTFL1. As shown by the correct molecular weight in HeLa cells, zebrafish LZTFL1 was expressed correctly *in vitro*. Furthermore, HeLa cells were transfected with Myc-tagged zebrafish LZTFL1, fixed, and probed with FITC-anti Myc antibodies. Zebrafish LZTFL1 showed predominant localization in the cytoplasm similar to that of human LZTFL1 (Wei et al., 2010).

Zebrafish LZTFL1 showed high identity with that of mammals and displayed a typical leucine zipper and coil–coil domain that are conserved among vertebrates, suggesting that zebrafish LZTFL1 may function as a negative regulator, suppressing HH pathway, similar to mammalian LZTFL1s (Seo et al., 2011; Wei et al., 2015). Subsequently, the corresponding luciferase reporter assay in the present study indicated that the ectopic expression of HeLa cells inhibited Gli1-luciferase activity. Together with the molecular characterization of the leucine zipper and coil–coil domain within zebrafish LZTFL1, this finding strongly suggested that zebrafish LZTFL1 was biologically functional and served as a negative regulator of the HH pathway.

Taken together, the full-length of zebrafish LZTFL1 cDNA was identified, and the protein structure was verified. Further subcellular distribution and functional characterizations confirmed that zebrafish LZTFL1 served as a modulator in the reduction of the HH pathway. These findings suggested the evolutionary conservation of the HH regulatory mechanism through LZTFL1 from fish to mammals. These results may enhance our understanding of the LZTFL1 function by using zebrafish model.

## Data Availability

The raw data supporting the conclusions of this manuscript will be made available by the authors, without undue reservation, to any qualified researcher.

## Ethics Statement

Animal experimental procedures were performed in accordance with the Regulations for the Administration of Affairs Concerning Experimental Animals approved and authorized by Zhejiang University (Document ID: 2018320). All applicable international, national, and/or institutional guidelines for the care and use of animals were followed.

## Author Contributions

QW made substantial contributions to the conception and design, analysis and interpretation of data, and drafting and revising the article. YC made contribution to the analysis and interpretation of data. Y-FG made contribution to the conception and design, drafting and revising of the article, and supervision of the study. WZ supervised the study.

## Conflict of Interest Statement

The authors declare that the research was conducted in the absence of any commercial or financial relationships that could be construed as a potential conflict of interest.

## Footnotes

1 http://pfam.xfam.org/
